# Non-specific low back pain in primary care in the Spanish National Health Service: a prospective study on clinical outcomes and determinants of management

**DOI:** 10.1186/1472-6963-6-57

**Published:** 2006-05-17

**Authors:** Francisco M Kovacs, Carmen Fernández, Antonio Cordero, Alfonso Muriel, Luis González-Luján, María Teresa Gil del Real, the Spanish Back Pain Research Network

**Affiliations:** 1 Departamento Científico, Fundación Kovacs, Palma de Mallorca, Spain; 2 Centro de Salud de Valldargent, Palma de Mallorca, Spain; 3 Centro de Salud de Formentera, Formentera, Baleares, Spain; 4 Unidad de Bioestadística Clínica, Node of R_MBEresearchnetwork(G03/90), Hospital Ramón y Cajal, Madrid, Spain; 5 Centro de Salud de Paterna, Paterna, Valencia, Spain; 6 Departamento Científico, Fundación Kovacs, Madrid, Spain

## Abstract

**Background:**

The Spanish National Health Service is a universal and free health care system. Non-specific low back pain (LBP) is a prevalent disorder, generating large health and social costs. The objectives of this study were to describe its management in primary care, to assess patient characteristics that influence physicians' decisions, and to describe clinical outcome at 2 months.

**Methods:**

A cross-sectional sample of 648 patients with non-specific low back pain was recruited by 75 physicians (out of 361 – 20.8%) working in 40 primary care centers in 10 of the 17 administrative regions in Spain, covering 693,026 out of the 40,499,792 inhabitants. Patients were assessed on the day they were recruited, and prospectively followed-up 14 and 60 days later. The principal patient characteristics that were analyzed were: sex, duration of the episode, history of LBP, working status, severity of LBP, leg pain and disability, and results of straight leg raising test. Descriptors of management were: performance of the straight leg raising test, ordering of diagnostic procedures, prescription of drug treatment, referral to physical therapy, rehabilitation or surgery, and granting of sick leave. Regression analysis was used to analyze the relationship between patients' baseline characteristics and physicians' management decisions. Only workers were included in the models on sick leave.

**Results:**

Mean age (SD) of included patients was 46.5 (15.5) years, 367 (56.6%) were workers, and 338 (52.5%) were females. Median (25th–75th interquartile range) duration of pain when entering the study was 4 (2–10) days and only 28 patients (4.3%) had chronic low back pain. Diagnostic studies included plain radiographs in 43.1% of patients and CT or MRI scans in 18.8%. Drug medication was prescribed to 91.7% of patients, 19.1% were sent to physical therapy or rehabilitation, and 9.6% were referred to surgery. The main determinants of the clinical management were duration of the episode and, to a lesser extent, the intensity of the pain (especially leg pain), a positive straight leg raising test, and degree of disability. The main determinant of sick leave was the degree of disability, followed by the characteristics of the labor contract and the intensity of leg pain (but not low back pain). After at least 2 months of treatment, 37% of patients were still in pain and approximately 10% of patients had not improved or had worsened.

**Conclusion:**

Although the use of X-Rays is high, determinants of physicians' management of LBP in primary care made clinical sense and were consistent with patterns suggested by evidence-based recommendations. However, after 2 months of treatment more than one third of patients continued to have back pain and about 10% had worsened.

## Background

Non-specific or common low back pain is defined as pain between the costal margins and the inferior gluteal folds, usually accompanied by painful limitation of movement, often influenced by physical activities and posture, and which may be associated with referred pain in the leg. Diagnosing common low back pain implies that the pain is not related to conditions such as fractures, spondylitis, direct trauma, or neoplastic, infectious, vascular, metabolic, or endocrine-related processes [1,2]. Low back pain is one of the most frequent ailments in industrialized countries, with a lifetime prevalence of more than 70% [3-5]. It is responsible for a major portion of work absenteeism [2,6,7] and is actually among those conditions which generate the greatest expense due to health and labor costs [2,5,8].

A considerable number of clinical guidelines have been developed for the management of acute and subacute patients [9,10]. The most commonly recommended approach to managing low back pain in primary care is the so called "diagnostic triage" [2,9,10]. Essentially, this consists of searching for the existence of signs or symptoms requiring referral to surgery or suggesting that the pain may be due to potential underlying specific causes. Those patients in which such "red flags" are identified are referred to surgery or to the appropriate diagnostic procedures. Those with no "red flags" are diagnosed as having "common" (or "nonspecific") low back pain, treated directly with no further examinations, and reassessed after 2 to 6 weeks[2,9-13].

There is considerable variation in LBP related clinical practice [14-17]. Most studies on what constitutes routine clinical practice for low back pain have been developed in Northern Europe, the United Kingdom and the United States [17-22]. The determinants of disability in the Mediterranean-Latin environment have proved to be different from those in Anglo-Saxon and Scandinavian countries [23]. Few data are available on clinical practice in Southern European countries [15], and those that are available in the Spanish National Health Service refer only to a specific local area [14].

The Spanish National Health Service is a public organization in which all health care services are provided free to every citizen. Physicians are free to prescribe, apply or refer their patients to any diagnostic procedure or treatment that they consider appropriate. That treatment will be provided to the patient for free as long as it is within the list of National Health Service accepted procedures. That is the case for virtually all commercially available drugs, diagnostic procedures, and non-pharmacological treatment, except for cosmetic surgery and some dental procedures. Primary care physicians receive no incentive to prescribe any diagnostic test or treatment, or to refer patients to any specialist. Actually, the primary care physician acts as a "gatekeeper" of access to specialized medical care and is responsible for deciding whether or not sick leave is granted. However, the patient may challenge the physician's decision, so that the decision is usually taken by consensus between the patient and the physician.

Identifying the clinical variables on which physicians base their decisions is as important as describing usual clinical practice and its variability. Additionally, the clinical course of subjects with low back pain treated in the Spanish National Health Service is actually unknown. Therefore, the objectives of this study were to describe the management of LBP in primary care in the Spanish National Health Service, to assess those patient characteristics that influence physicians' decisions, and to describe clinical outcome at 2 months.

## Methods

### Study population

The directors of 40 Primary Care Centers of the Spanish National Health Service were sent a letter inviting them to join the study. Those Centers were those in which there was at least one physician having expressed or stated an interest at any time on any kind of research on back pain. The Centers were located in 10 of the 17 Spanish administrative regions and they covered a total population of 693,206 subjects, out of a total Spanish population of 40,499, 792 inhabitants [24]. The Directors of the Centers were asked to forward the invitation to the 361 physicians working in those practices, and 94 physicians (26.0%) accepted to participate in the study, although in the end only 75 (20.8%) actually recruited patients.

Primary care physicians were asked to recruit all subjects who visited them because of low back pain, with or without leg pain, 18 years of age or older. Exclusion criteria were functional illiteracy (inability to answer the questionnaires used to assess pain and functional disability), illnesses affecting the central nervous system, and "red flags" for surgical referral (saddle anesthesia, recent onset of bladder dysfunction or anal sphincter impairment, major or progressive motor weakness, sensory level, or widespread neurological signs), or for possible specific spinal pathology (oncologic disease during the previous 5 years, constitutional symptoms-unexplained weight loss, fever, chills-, recent urinary tract infection, history of intravenous drug use, or immunocompromised host)[2]. The study protocol was approved by the institutional review boards of the participating centers and all patients gave written informed consent for use during a 6-month period of his/her data regarding severity of symptoms, health care, and sick leave for low back pain.

This being an observational study, there was no intervention per se. Physicians were given no instructions, but encouraged to treat their patients as they usually do, and no attempt was made to homogenize their practice. They were told that the objective of the study was to contribute to the knowledge of low back pain. Management of non-specific low back pain in the primary care setting of the Spanish National Health Service is based on clinical history, physical examination, recommendation of diagnostic tests, medical counseling, drug treatment, physical therapy, rehabilitation, or referral to specialists. All visits of patients and all the diagnostic procedures, treatments, and referrals that were prescribed by the primary care physicians were registered in structured questionnaires.

### Outcome assessment

The clinical condition of each patient was assessed at the primary care center by his/her general practitioner on the day of inclusion in the study (baseline assessment, day 1) and 15 and 60 days later. Besides these three obligatory follow-up assessments, all visits requested by the patient during the study period because of low back pain were considered additional voluntary visits and data on patients' clinical evolution and on physicians' management of the patients were recorded.

At baseline, the following variables were recorded: age (date of birth); sex; educational level (classified in 5 categories, from lower to higher level); duration of pain (number of days elapsed since the current episode of pain appeared, when the patient was recruited), chronicity of pain when entering the study (classified as acute, subacute or chronic, and defining subacute as a duration of pain between 14 and 90 days) [23,24]; previous episodes of low back pain (zero, one or two, more than two); current use of drug treatments (yes/no), previous spinal surgery in the dorsolumbar region (number of procedures); living alone, living with someone or institutionalized; usual job; and the number of plain radiographs, CT scans, MRI studies, neurophysiologic or other diagnostic tests, treatments, and referrals to specialists or visits to the emergency room because of low back pain during the 3 months prior to entering the study.

At each assessment, including the first one, the following variables were recorded: current pregnancy (yes/no); work status (potentially active-self-employed or employed by others- or not potentially active-retired, unemployed, student, housewife, other-), sick leave (no, yes-number of days-, not applicable), reason for sick leave (low back pain, other, not applicable – e.g. unemployed population-), severity of low back and leg pain (assessed by means of independent visual analogue scales (VAS) [26] in which a higher score means a more severe pain, on a 0–10 range), degree of functional disability (assessed by the previously validated Spanish version of the Roland Morris questionnaire [27] in which a higher score reflects a higher level of disability, on a 0–24 range), and quality of life level (measured by the EuroQol questionnaire [28], in which a higher score reflects a better quality of life on a 0 to 1 range), number of physician visits by the patient throughout the period (to primary care physicians, public specialists, private specialists and alternative medicine), diagnostic tests (X-rays, CT scans, MRI studies, electromyography, blood tests, other) and drug treatment which were prescribed by the primary care physician, and referrals to rehabilitation, physical therapy, orthopedic surgery, neurosurgery or other services. At each assessment, a straight leg raising test (SLR) was performed at the discretion of the primary care physician (and classified as <30°, between 30° and 60°, and >60°). Results of the SLR test were based on the range of motion triggering referred pain to the leg.

"Key descriptors" of the essential aspects of clinical practice in low back pain were defined, and this information was collected in each of the assessments. Performing the SLR test at any moment between the first and third assessments (considering the SLR and the crossed SLR equally [29]) was considered as descriptive of the physical examination. The ordering of imaging and neurophysiologic tests at any moment during the study was considered as descriptive of the request for diagnostic tests. Imaging tests were classified as X-rays, or CT scan/MRI, since in some regions the prescription of one or the other depends solely on its availability. Additionally, since in some regions the primary care physician can only prescribe the CT scan or MRI via the orthopedic surgeon or the neurosurgeon, referrals to those specialists with such intention were also considered as prescriptions for a CT scan or MRI. Since a recent history of diagnostic testing can influence its prescription, the realization of these tests was taken into account from the time of recruitment to three months prior.

The prescription of medication in the first visit (NSAIDs, muscle relaxants, analgesics, and "other") or referral (to physical therapy, rehabilitation, orthopedic surgery, neurosurgery, or "other") at any moment during follow-up were considered descriptive of treatment. Physician's counsel was not included as descriptive of treatment because it was understood to be inherent to all medical practice, and because no validated system to classify its content was found ("active management", "postural hygiene", etc.).

The granting of sick leave by the general practitioner at any moment between the first and third assessments was considered descriptive of sick leave.

Data recording and transference to the database were as follows: questionnaires on pain, disability and quality of life were self-administered and completed by the patient on his/her own in the absence of the treating physician, other health care staff, family members, or accompanying persons. Completed self-reported instruments were then given to the auxiliary staff of the primary care center who stapled scales and questionnaires to the patient's data collection form. At the end of the working day, ratings of the scales were transcribed to the data collection form by the treating physician. At each Primary Care Center, concordance of scores in the data collection form and ratings of self-administered instruments were verified by the study coordinator in the Center. Structured data collection forms with information on patients' clinical management were then sent along with patients' self-reported instruments to the central coordination office, where data were entered in a database by two administrative assistants who double-checked data entry. They also double checked that scores on pain, disability and quality of life scales coincided with actual patients' ratings of the VAS, Roland-Morris and EuroQol questionnaires. All throughout the process, patients' answers on the self-administered questionnaires were considered the gold standard, but no inconsistencies were actually found.

### Analysis

The analysis was done by a team of biostatisticians who had no connection with the primary care physicians involved in this study. Frequencies were calculated for categorical variables. For continuous ones, means and standard deviations were calculated for those with a normal distribution, and median and interquartile ranges for the rest. Logistic regression models were developed to explore the influence of different factors on the key descriptors of clinical management of non-specific low back pain. In each model the descriptor was used as a dependent variable. Colinearity of the maximal models was evaluated with the criteria proposed by Belsley [30]. A backward strategy was used, using the value *p *< 0.05 to eliminate variables from the model.

The following variables were introduced in all maximal models: sex, previous number of episodes of low back pain when entering the study (recoded as "less than three" and "three or more"), intensity of lumbar and leg pain at the first assessment, degree of disability at the first control, and chronicity of pain when entering the study (acute, subacute or chronic) [23,25]. The variable SLR was also included in all models, categorized as not done, negative (≥60°) or positive (<60°), except in the model that referred to doing the test. Only workers were included in the model on sick leave. Since in the Spanish Social Security System self-employed persons are economically penalized while on sick leave, as opposed to those employed by third parties, workers were classified as "self employed" or "employed by another". In all other models patients in different work situations were included, and the variables "work status" (recoded as "potentially active" and "other") and "sick leave" ("yes" or "no") were included.

## Results

During an 18-month period, 653 patients were recruited, of which 5 were excluded at the first assessment (one for low back pain that was secondary to a traumatism, another for possible infectious origin, and three for sciatica with progressive motor weakness). At the second assessment two patients were excluded (one because of a systemic disease and the other due to a house move), and at the third assessment another two (one for a direct traumatism to the spine, and the other for having suffered an illness affecting the central nervous system). Thus, a total of 648 patients fulfilled the inclusion criteria at the first assessment and were included in the study, and 374 (57.7%) completed all assessments.

The mean (standard deviation, SD) number of patients recruited by each physician was 8.65 (8.5). Table 1 indicates the characteristics of patients included in the study. As seen in that table, median (25th–75th interquartile range) duration of pain when entering the study was 4 (2–10) days, and only 28 patients (4.3%) were chronic. A total of 367 patients (56.6%) were in a potentially active work situation.

A total of 495 patients came for the second visit and 374 came for the third, and there were no differences in baseline data between patients who attended and those who did not attend those assessments (data not shown). Table 2 shows the results of the assessments. Two months after the first visit, low back pain had disappeared in 35.6% of patients, and leg pain in 44.8% of those who previously had it (Table 2). However, at that time at least 37% of patients still had low back pain, which had increased in 6.7% of them (mean increase of 2.2), while leg pain had increased in 11.7% of patients who previously had it (mean increase of 1.8). Leg pain had appeared at 14 days in 12.9% of patients who had no leg pain at the first assessment, and at 60 days in 13.5%.

In comparison with baseline data, Roland Morris disability scores improved by 5.0 points at 14 days and by 6.6 points at 60 days. However, they remained the same in 8,7% of patients at 14 days, and in 4.3% at 60 days, and worsened in 10.5% of patients at 14 days and in 7.3% at 60.

Throughout the study, physicians performed the SLR on 439 patients (67.7%), requested imaging tests for 279 (43.1%) and neurophysiologic tests for 21 (3.2%), prescribed medication at the first visit for 594 (91.7%) -mostly non-steroidal anti-inflammatory drugs, analgesics and muscle relaxants-, referred 124 (19.1%) to physical therapy or rehabilitation, and 62 (9.6%) to orthopedic surgery or neurosurgery (Table 1).

During the study period, 175 of the 367 (47.7%) patients in a potentially active work situation went on sick leave (Table 3). Physicians granted sick leave to 158 patients at the first assessment (43.8% of those in whom sick leave was possible). Of these, 125 (79.1%) were still on sick leave for that reason at 14 days, and 43 (27.2%) at 60 days. Some patients who were not on sick leave at the baseline assessment were on sick leave at 14 and 60 days (Table 2).

In addition to the planned visits, 22.8% of patients requested medical attention in primary care during the first two weeks, 8.7% visited other health services, and 3.2% went to the emergency room. Between 2 weeks and 2 months, 21.7% of the patients requested additional health care in primary care, 13.4% visited other specialists, and 2.4% went to the emergency room. Between 2 and 6 months, only 23 (6.1%) patients requested health care in primary care, and 8 of them visited a specialist (orthopedic surgeon or neurosurgeon).

Table 4 shows the "key descriptors" of clinical practice separately for acute, subacute and chronic patients. The low number of patients for whom neurophysiologic tests were requested did not permit logistic regression models on this variable. The results of the regressions are shown in Table 4. The only variable associated with performing the SLR is the intensity of leg pain. The prescription of X-rays was associated with having previously had more than 2 episodes of low back pain, longer duration of the current episode, greater degree of disability, greater intensity of leg pain, and greater limitation in the SLR test. The same variables that were associated with plain radiographs were also associated with the prescription of CT scans or MRI studies, except for the existence of previous episodes of low back pain (which was irrelevant), and with the addition of sex (OR = 1.9 for referral in males *vs*. females). The prescription of medication was associated with greater limitation in the SLR and shorter duration of pain. The referral to rehabilitation or physical therapy was associated with longer duration of pain, greater intensity of low back pain, more than 2 previous episodes of low back pain, and greater limitation in the SLR. Referral to a surgeon (orthopedic or neurosurgeon) was associated with a greater intensity of leg pain, greater level of disability, longer duration of pain, and being male rather than female (OR = 1.9). Sick leave was associated with greater severity of leg pain, being employed by another (as opposed to self-employed) and especially with the level of functional disability (Table 4).

## Discussion

The results of this study reflect clinical practice for non-specific low back pain in the primary care setting of the Spanish National Health Service. In accordance with these results, the probability of prescribing X-Rays increases when the patient has had more than 2 previous episodes, the duration of the episode is longer, and disability, intensity of leg pain (but not low back pain), and limitation in the SLR are greater. The same variables influence the prescription of MRI studies, except that this is not influenced by the number of previous episodes and that the probability of prescription is also greater for men than for women. This is in accordance with what has been described in other settings, [31] and might be related to the different experience of pain and body awareness in women [2].

The probability of prescribing medication increases with a positive (<60°) SLR test and with having acute (vs. chronic) pain (Table 4). This last association is important, because a longer duration of the episode is associated with a lesser probability of a prescription for medication and a greater one for referral to physical therapy or rehabilitation. Intensity of back pain did not show an association with a greater probability of prescriptions for medication probably because all of the patients in this study had pain of sufficient intensity to warrant a doctor's visit, and 90% of the patients were placed on medication (Tables 1 and 3).

In addition to a longer duration of the episode, the probability of referral to physical therapy or rehabilitation is also greater when low back pain is more intense, when there is greater limitation in the SLR, and when the patient has had previous episodes of low back pain. Factors associated with a greater probability of referral to surgery are being male, having a greater severity of leg pain, having a greater level of disability and, especially, that the episode is chronic (Table 4).

The apparent relationship between the granting of sick leave and being employed by another (vs. self-employed) is not surprising since in the Spanish Social Security System only self-employed workers have an economic incentive to continue working. However, it is important to point out that the level of disability and the intensity of leg pain (but not low back pain) are more closely related to the granting of a sick leave, which suggests that physicians do not consider sick leave a treatment for low back pain, and only grant it when the level of disability requires it.

The main common recommendations from the different currently available evidence based practice guidelines for acute non-specific low back pain are: avoiding bed rest and trying to stay active (which includes not systematically granting sick leave), prescribing drug treatment (non-steroidal antiinflamatory drugs and analgesics, eventually together with muscle relaxants) only for limited periods when the pain exacerbates, and restricting imaging procedures and referral to surgery to a subset of patients [9-13]. In this study, the criteria that have proven to influence physicians' practice are generally consistent with those recommendations and make clinical sense. They limit drug treatment to acute phases, shifting to exercise and rehabilitation when pain is prolonged, they don't systematically prescribe imaging tests or grant sick leave, and they only refer to surgery the most chronic cases showing a greater degree of disability, and in which leg pain is more intense [32,33].

However, although factors associated with X-ray prescription make clinical sense, the rate of prescription was 43.1%, which is higher than recommended [9-13]. Most factors influencing the prescription of plain radiographic studies overlap with those related to CT scan or MRI prescription. Since in some regions primary care physicians cannot prescribe the latter without referring the patient to the specialist, it is possible that they use plain radiographs as a first step. The over prescription of X-rays might also be due to physicians overestimating their diagnostic value or, most likely, to their desire to meet the patient's expectations. In addition, the first guideline on LBP available in Spain was issued after this study was done, [11-13,34] and no effort had been made before that to reduce the inappropriate prescription of X-Rays for LBP in primary care.

Although the design and objective of the study were not to compare clinical practice in different regions, and the study population was too small for that purpose, in general the criteria for management by family physicians were consistent throughout the different Spanish regions (data not shown). The main difference between regions was in the use of muscle relaxants, a subject on which scientific evidence is partial [35]. In fact, a previous Spanish study done in a small geographical area showed the prescription of muscle relaxants to be greater than the mean rate of use seen in this study [14]. However, in the present study the global rate of use of pharmacological treatment was very similar in the different regions, and coincides with what was found in that study.

The mean evolution of pain and disability was favorable, as was to be expected in acute patients due to regression toward the mean, to the natural course of the condition or to the effect of treatment. However, 64.4% of patients assessed at two months continued to have pain or disability, and in approximately 10% of them those variables had not improved at all, or had even worsened. Even if all the patients who did not comply with the follow-up assessments had been totally asymptomatic, patients with pain or disability at two months represent 37% of those seen initially. This is consistent with the results of other studies [36-43], and reflects the fact that although the prognosis of acute low back pain may be favorable in many cases, there is a subgroup of patients in which that tendency is different, in spite of the treatment that is followed. Fourteen days after the appearance of pain, the episode becomes subacute [25]. After that period, pain and disability become the main determinants of quality of life, and the risk of pain becoming chronic sharply increases [23,25]. This suggests that treatments that have proven to be effective for the improvement of pain and disability should be applied as soon as possible after that period [11-13,44].

The degree of disability and the intensity and duration of low back pain have proved to be the main determinants of quality of life in Spanish patients with non-specific low back pain [23,25,45]. Therefore, this study suggests that there was an important decrease in the quality of life of those patients in which pain and disability did not improve throughout the study. In this study, quality of life was measured with the Spanish version of EuroQol [28]. However, this version is inaccurate to predict quality of life in Spanish LBP patients, since it underestimates it in 211 out of 243 possible health states, whereas it overestimates it in the rest of them [46]. It is thus impossible to accurately determine the evolution of the quality of life of patients in this study.

There are other limitations to this study. Fear-avoidance beliefs (FAB) on low back pain were not recorded, since there were no validated instruments in Spanish for that purpose when it was performed. However, contrary to what has been shown in Northern Europe and Anglo-Saxon countries, the influence of FAB on disability and quality of life is virtually irrelevant in Spanish patients [45]. Therefore, it is not likely that FAB influenced these results.

Only 20.8% of the primary care physicians invited to participate in the study did so. It is possible that they were the physicians most interested in low back pain and possibly the most up-to-date on the management of this condition. If that were so, the overall management of low back pain in the Spanish National Health Service could be more inconsistent with evidence-based recommendations. However, physicians working in the Spanish National Health Service do not have any incentive to participate in studies during their work time, including this one, and only do so because of their own scientific interest. Their rate of participation in this study is similar to that found in other reports[44]. Additionally, when the study was done no clinical guideline on LBP management was available in Spain, the physicians had not followed any specific post-graduate training on LBP management, and no steps were taken to homogenize their criteria. Moreover, they were from 10 of the 17 administrative Spanish regions, they had no contact between them before the study began, and they did not relate to clinical management criteria during the planning or execution of the study. All of the above suggests that participants' management of LBP can be considered as fairly representative of routine practice in the primary care of the Spanish National Health Service. In fact, the clinical management described in this study is consistent with what was observed in all of the physicians working in a reduced geographical setting [14].

Because the recruitment for this study was cross-sectional, patients with acute, subacute, and chronic low back pain could have been included. However, only 28 patients had chronic low back pain (4.3% of the sample). Since it was anticipated that the duration of pain may influence clinical management, patients' chronicity when entering the study was included in all the regression models. In fact, the results of this study show that the ordering of X-ray, CT scans or MRI studies, the prescription of medication, and the referral to rehabilitation, physical therapy, and surgery, are different for acute, subacute and chronic patients.

Only 374 patients (57.7% of those included) were seen at all assessments. The Spanish National Health Service is universal and free, and patients with non-specific low back pain tend to visit their physician when they feel pain. However, once they are asymptomatic they stop their visits, as has been shown in previous studies on low back pain performed in Spain, in which recall mechanisms were used [44]. This may be consistent with the scarce influence that fear avoidance beliefs exert on Spanish patients [45], reflecting that they do not tend to over-medicalize the process, but instead resume their normal activity as soon as the pain allows. In this study no recall mechanisms were planned because the intention was to minimize the variations of normal conditions of usual clinical practice, and because the objective was not to study the evolution of patients throughout a period of time but the determinants of the physicians' clinical practice.

## Conclusion

This study shows that the main variables affecting physicians' management of non-specific low back pain in the primary care setting of the Spanish National Health Service were duration of the episode and, to a lesser extent, intensity of low back pain, especially of leg pain, limitation in the SLR, and degree of disability. Although the rate of use of X-ray examinations was high, the criteria for management of non-specific low back pain were consistent with evidence-based recommendations. However, 2 months after treatment, more than one third of patients continued to have pain and in about 10%, the outcome was poor.

## Abbreviations

CI: confidence interval

CT: computed tomography

MRI: magnetic resonance imaging

OR: odds ratio

SLR: straight leg raising test

VAS: visual analogue scale

## Funding

This study was funded by the Kovacs Foundation and Fondo de Investigación Sanitaria (grant number 98/1264), Madrid, Spain.

## Competing interest declaration

The author(s) declare that they have no competing interests.

## Authors' contributions

Francisco M. Kovacs is the principal investigator, participated in the conception, design and development of the study, writing of the article, and editorial decisions.

Carmen Fernández participated in the development of the study and in data collection.

Alfonso Muriel participated in data analysis and in the writing of the article.

Luis González-Luján participated in the development of the study and in data collection.

María Teresa Gil del Real participated in the design, development of the study and writing of the article.

Other members of the Spanish Back Pain Research Network participated in the development of the study, data collection, data analysis, and the writing of the article.

## Note

Other members of the Spanish Back Pain Research Network who participated in the study are: V. Abraira and J. Zamora, Hospital Ramón y Cajal, Madrid; M. Gestoso, N. Mufraggi, M. Martín, and S, Santos, Fundación Kovacs, Palma de Mallorca; V. González, L. Palacios, J. Mardones, J. A. Estévez, and E. Marzo, Centro de Salud de Albia, Bilbao; A. Fajardo and I. Barrio, Centro de Salud de Cadalso, Madrid; P. González and B. Navalón, Centro de Salud de Los Castillos, Madrid; M.A. Usero, M. Arnalte, and M. Rojo, Centro de Salud de Perales del Río, Madrid; C. Isanta and N. Giménez, Centro de Salud de Perpetuo Socorro, Huesca; P. Puente, Centro de Salud de San Agustín, Burgos; A. Careaga, B. Amutxategui, M. Fernández, and J. M. Gendive, Centro de Salud de Casco Viejo, Bilbao; R. Ruiz and M. Chaparro, Centro de Salud de Barconcillo, Guadalajara; S. Borregón, J.E. Villares, and A. Ruiz, Centro de Salud de las Ciudades, Madrid; M. Gómez, L. Perelló, J. Ramón, J. Ripoll, and C. Mateu, Centro de Salud de Son Serra/La Vileta, Palma de Mallorca; P. Ibáñez and A. Jover, Centro de Salud de Arquitecto Bennassar, Palma de Mallorca; S. Segura, E. Viñuelas, I. Sanz, and M. Delgado, Centro de Salud de Carrascosa del Campo, Cuenca; M. C. Vargas and M^a ^Luz Besteiro, Centro de Salud de Guayaba, Madrid; C. Tourné and L. V. Marín, Centro de Salud de Abanilla, Murcia; E. Thomás, M. J. Nadal, and A. Sánchez and G. Rodríguez, Centro de Salud de Jazmín; J.C. Vergara, F. Gordillo, and L. C. Morales, Centro de Salud de Pintores, Madrid; E. Arranz and A. B. Bellón, Centro de Salud de Isabel II, Madrid; A. Martín, D. Giménez, and N. Herranz, Centro de Salud de Eras del Bosque, Palencia; C. Rubio, Centro de Salud de Plaza Argel, Cáceres; R. Elena Duro, S. Ribot, J. Coll, V. Reyes, and M. Arrate Olivera, Centro de Salud de de Coll d'en Rebassa, Palma de Mallorca; R.A. Escribá, H. Moreno, and J. Rubio, Centro de Salud de Palomares, Madrid; J. Gili, Centro de Salud de Santa Catalina, Palma de Mallorca; J. Martín, M. J. Santero, and L. Cossio, Centro de Salud de Aranjuez II, Madrid; A. de Torres, Centro de Salud de Pinto, Madrid; J. C. García and D. Jiménez, Centro de Salud de Alcolea del Pinar, Guadalajara; R. Martín, Centro de Salud de los Cármenes, Madrid; V. Portuguéz, Centro de Salud de UB de Jesús, Ibiza; E. Fernández, I. Gil, J. Moreno, A. Aura, R. Vercher, G. Albert, and G. Díaz, Centro de Salud de Salvador Allende, Valencia; P. Pascual, M. Llinás, and J. Ripoll, Centro de Salud de Valldargent, Palma de Mallorca; E. Rodríguez, J. González, and C. Nicolau, Centro de S'Escorxador, Palma de Mallorca; M. A. Castillo, Centro de Salud de Puerta Bonita, Madrid; R. Llidó, Centro de Salud Santa Eulalia, Ibiza; S. Luna, B. Leal, and J. Nieto, Centro de Salud de Alburquerque, Badajoz; C. Santos, Centro de Salud de C'n Misses, Ibiza; L. M. Rubiera, I. Aguilar, M. D. San Román, C. Sánchez, and U. López, Centro de Salud de Guzmán el Bueno, Madrid; and J. Llobera, Unidad de Investigación, Gerencia de Atención Primaria, Ib-Salut, Palma de Mallorca, Spain.

## Pre-publication history

The pre-publication history for this paper can be accessed here:



## Figures and Tables

**Table 1:** Data of patients included in the study (n = 648).

Data	Number (%)	Mean (SD) or median (25th–75th range)
Sex		
Female	338 (52.2)	
Male	310 (47.8)	
Age, mean (SD)		46.45 (15.5)
Education level		
No studies	14 (2.1)	
Did not finish basic studies	153 (23.6)	
Basic studies	205 (31.6)	
Medium-level studies	162 (25.0)	
Higher level studies	97 (15.0)	
Missing	17 (2.6)	
Living conditions		
Living alone	57 (8.8)	
Living with someone	500 (77.2)	
Institutionalized	5 (0.8)	
Missing	86 (13.2)	
Severity of low back pain (VAS); mean (SD)		5.89 (2.2)
Leg pain		
Yes	383 (59.1)	
Severity of leg pain (VAS); median (range)		4.3 (2.6–6.7)
Disability (Roland Morris Questionnaire); mean (SD)		11.30 (5.5)
Quality of life (EuroQol); mean (SD)		0.49 (0.2)
Previous episodes of low back pain		
None	209 (33.5)	
1 or 2	180 (28.8)	
3 or more	235 (37.7)	
Duration of current episode (days); median (IQR)		4 (2–10)
Chronicity		
Acute	500 (77.2)	
Subacute	118 (18.2)	
Chronic	28 (4.3)	
Patients visiting the recruiting physician for the first time	393 (60.6)	
Patients visiting the recruiting physician at follow-up	255 (39.4)	
Diagnostic tests	112 (17.3)	
X-rays on the first visit	101 (15.6)	
CT scan on the first visit	14 (2.2)	
MRI on the first visit	16 (2.5)	
Neurophysiological tests on first visit	9 (1.4)	
Visits to emergency room	64 (9.9)	
Working status:		
Housewife, retired, other	281 (43.4)	
Workers	367 (56.6)	
Self-employed	58 (8.9)	
Employed by others	309 (47.7)	
Being on sick leave	158 (24.4)*	
SLR test		
Not done	243 (37.5)	
< 30	88 (13.6)	
30–60	119 (18.4)	
> 60	198 (30.6)	
Prescription of medication^†^	594 (91.7)	
NSAIDs	370 (57.1)	
Analgesics	212 (32.7)	
Muscle relaxants	97 (15)	
Corticoids	21 (3.2)	
Gastric protectors	14 (2.2)	
Anxiolitics	30 (4.6)	
Referrals	62 (9.6)	
Orhopedic surgery	34 (54.8)	
Rehabilitation	17 (27.4)	
Neurosurgery	5 (8.1)	
Other	11 (17.7)	

*Sick leave was only possible to check among the 367 workers included in the study. The 158 subjects on sick leave represent 43.07% of them.

^†^Percentage of individuals using each kind of medication treatment among those using medication treatment.

**Table 2:** Status of patients at the 2nd and 3rd visits

Data	2nd visit (n = 495)	3rd visit (n = 374)
Low back pain (yes), n (%)	397 (80.2%)	241 (64.4%)
Severity of low back pain, VAS, mean (SD)	3.69 (2.3)*	3.24 (2.3)*
Leg pain (yes), n (%)	249 (50.3%)	150 (40.1%)
Leg pain in those who had it at first visit, n (%)	225 (72.8%)	132 (55.2%)
Severity of leg pain (VAS), median (range)	3.0 (1.1–5.2)*	3.0 (1.3–4.7)*
Functional disability, mean (SD)	6.24(5.6)	4.75 (5.2)
Quality of life, mean (SD)	0.63 (0.2)	0.69 (0.2)
SLR, n (%)		
Not done	308 (62.2)	272 (72.4)
< 30°	56 (11.3)	37 (9.9)
30–60°	49 (9.9)	21 (5.6)
> 60°	82 (16.6)	44 (11.8)
Sick leave, n (%)^†^	140 (49.1)	51 (24.6)

*Includes only patients who had that type of pain at the corresponding assessment.

^†^Percentages refer to the 367 patients in a potentially active work situation, in which sick leave was possible.

**Table 3:** Overall prescription rate

	Acute n = 500	Subacute n = 118	Chronic n = 28
Medication prescribed at first visit, no (%)	459 (91.8)	111 (94.1)	22 (78.6)
NSAIDs	287 (57.4)	69 (58.5)	13 (46.4)
Analgesics	167 (33.4)	39 (33.1)	6 (21.4)
Muscle relaxants	79 (15.8)	15 (12.7)	3 (10.7)
Corticoids	18 (3.6)	3 (2.5)	0 (0.0)
Gastric protectors	9 (1.8)	4(3.4)	1(3.6)
Tranquilizers	23 (4.6)	5 (4.2)	2 (7.1)
Other medication	23 (4.6)	4 (3.4)	1(3.6)
Procedures performed throughout the study, n (%)			
SLR	351 (70.2)	74 (62.7)	13 (46.4)
Plain radiographs	193 (38.6)	66 (55.9)	18 (64.3)
CT or MRI scans	75 (15.0)	32 (27.1)	14 (50.0)
Neurophysiologic tests	12 (2.4)	5 (4.2)	4 (14.3)
Referral to rehabilitation or physical therapy	83 (16.6)	33 (28.0)	7 (25.0)
Referral to orthopaedic surgery or neurosurgery	31 (6.2)	19 (16.1)	11 (39.3)
Sick leave	136 (50.6)*	25 (33.8)*	13 (59.1)*

*Percentages refer to the 367 patients in a potentially active work situation, in which sick leave was possible.

**Table 4:** Results of the logistic regression analysis

Descriptor	Variable	Odds ratio (95% CI)	*P *value	Likelihood ratio test	*P *of the test
SLR	Intensity of leg pain	1.3 (1.2–1.4)	0.000	55.2	0.000
X-rays	Intensity of leg pain	1.2 (1.0–1.2)	0.017	76.4	0.000
	>2 episodes of low back pain	1.9 (1.4–2.7)	0.000		
	Disability	1.1 (1.0–1.1)	0.002		
	Chronicity*		0.000		
	Subacute	2.3 (1.5–3.5)	0.000		
	Chronic	2.7 (1.2–6.1)	0.022		
	SLR		0.002		
	> 60°	0.7 (0.4–1.0)	0.038		
	< 60°	1.5 (1.0–2.2)	0.085		
CT scan or MRI	Sex (male)*	1.9 (1.3–3.0)	0.003	93.4	0.000
	Intensity of leg pain	1.1 (1.1–1.2)	0.001		
	Disability	1.1 (1.0–1.1)	0.000		
	Chronicity*		0.000		
	Subacute	2.7 (1.6–4.5)	0.000		
	Chronic	5.8 (2.5–13.6)	0.000		
	SLR		0.002		
	> 60°	0.7 (0.4–1.3)	0.230		
	< 60°	1.9 (1.1–3.3)	0.016		
Prescription of medication	Chronicity*		0.022	18.4	0.001
	Subacute	1.5 (0.6–3.4)	0.370		
	Chronic	0.3 (0.1–0.8)	0.013		
	SLR		0.003		
	> 60°	0.5 (0.3–1.0)	0.059		
	< 60°	2.2 (0.9–5.1)	0.073		
Referral to rehabilitation or physical therapy	Intensity of low back pain	1.1 (1.0–1.2)	0.015	29.5	0.000
	>3 episodes of low back pain	1.5 (1.0–2.3)	0.039		
	Chronicity*		0.006		
	Subacute	2.1 (1.3–3.4)	0.002		
	Chronic	1.9 (0.8–4.7)	0.172		
	SLR		0.018		
	> 60°	0.6 (0.4–1.1)	0.092		
	< 60°	1.4 (0.9–2.2)	0.184		
Referral to surgery (orthopedic surgery or neurosurgery)	Sex (male)	2.0 (1.1–3.5)	0.023	61.6	0.000
	Intensity of leg pain	1.2 (1.1–1.3)	0.002		
	Disability	1.1 (1.0–1.2)	0.001		
	Chronicity*		0.000		
	Subacute	3.5 (1.8–6.7)	0.000		
	Chronic	9.9 (4.0–24.4)	0.000		
Sick leave*	Roland-Morris	1.2 (1.1–1.2)	0.000	58.7	0.000
	Intensity of leg pain	1.1 (1.0–1.2)	0.024		
	Employed by others	2.0 (1.1–3.9)	0.031		

*The variables chronicity and SLR are coded as "dummy variables", using the categories "acute" and "not done", respectively, as reference. For "sex", the reference is the "female" category. Since sick leave was only possible in workers, the models on sick leave only included the 367 workers in the study.

For continuous variables, the table shows the OR of each one point increase in the corresponding scale. For dichotomous variables, the table shows the OR for each category
